# Al_2_O_3_ Particles on Titanium Dental Implant Systems following Sandblasting and Acid-Etching Process

**DOI:** 10.1155/2019/6318429

**Published:** 2019-06-02

**Authors:** Peter Schupbach, Roland Glauser, Sebastian Bauer

**Affiliations:** ^1^Schupbach Ltd, Laboratory for Histology, Electron Microscopy and Imaging, CH-8800 Thalwil, Switzerland; ^2^Cosmodent Clinic, CH-8001 Zurich, Switzerland; ^3^Material Research and Surface Technologies, Nobel Biocare Services AG, CH-8302 Kloten, Switzerland

## Abstract

Dental implants with moderately rough surfaces show enhanced osseointegration and faster bone healing compared with machined surfaces. The sandblasting and acid-etching (SA) process is one technique to create moderately rough dental implant surfaces. The purpose of this study was to analyse different commercially available implant systems with a SA-modified surface and to explore the widespread notion that they have similar surface properties regarding morphology and cleanliness. SA-modified surfaces of nine implant systems manufactured by Alpha-Bio Tec Ltd, Camlog Biotechnologies AG, Dentsply Sirona Dental GmbH, Neoss Ltd, Osstem Implant Co. Ltd, Institute Straumann AG, and Thommen Medical AG were analyzed using scanning electron microscopy (SEM) and energy dispersive X-ray spectroscopy (EDX) and examined for surface cleanliness. Six implants from three different lots were selected per each implant system. Mean particle counts for each implant and the mean size of the particles were calculated from three different regions of interest and compared using ANOVA and Tukey's test. SEM analysis showed presence of particles on the majority of analyzed implant surfaces, and EDX evaluations determined that the particles were made of Al_2_O_3_ and thus remnants of the blasting process. SPI®ELEMENT INICELL® and Bone Level (BL) Roxolid® SLActive® implant surfaces showed the highest mean particle counts, 46.6 and 50.3 per area, respectively. The surface of BL Roxolid® SLActive® implant also showed the highest variations in the particle counts, even in samples from the same lot. The mean size of particles was 1120±1011 *μ*m^2^, measured for USIII CA Fixture implants, while the biggest particle was 5900 *μ*m^2^ found on a BL Roxolid® SLActive® implant. These results suggest that not all manufacturers are able to produce implant surfaces without particle contamination and highlight that the surface modification process with the SA technique should be appropriately designed and controlled to achieve a clean and consistent final medical device.

## 1. Introduction

Dental implant surfaces play a key role in osseointegration and thus continue to drive biomedical research investigations on how surface modifications affect osteogenic potential [1]. In the first decades since their introduction by P-I Brånemark [2, 3], dental implants had primarily machined surfaces, which were created by milling, turning, or polishing techniques. Although machined implants demonstrate high long-term survival rates following osseointegration process [4, 5],* ex ante* they require a relatively long healing time of 3 to 6 months depending on the anatomical location and the quality of bone [6] and are characterized by a relatively high rate of early failures [7]. One hypothesis to account for these early failures is that machined surfaces have an insufficient surface roughness to promote osteogenic cell attachment and bone deposition to form enough bone-to-implant interface. Subsequent research has revealed that moderate surface roughness, i.e., Sa range of 1-2 *μ*m, provides optimal conditions to promote osseointegration [8]. In clinical studies, implants with moderately rough surfaces have demonstrated faster osseointegration and higher long-term survival rates compared with machined implants [4, 5, 9–12]. An increased surface area of moderately rough implant surfaces allows for better cell attachment, contact osteogenesis, and bone ingrowth, which result in improved implant stability and enable application of immediate and early loading protocols [13, 14].

**Figure 1:**
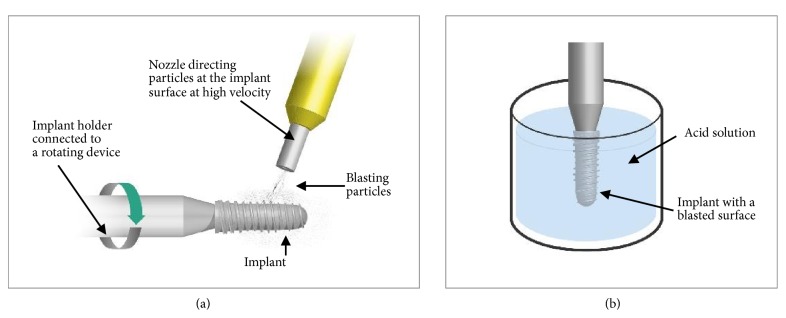
Schematic representation of the sandblasting (a) and acid-etching process (b).

Several processes to increase the surface roughness of titanium implant surfaces based on additive, subtractive, chemical, and electrochemical surface treatments can be used [1]. Major surface modification techniques include titanium plasma spraying, coating with hydroxyapatite, sandblasting, acid-etching, sandblasting combined with acid-etching, laser ablation, and anodization. Today, sandblasting and acid-etching (SA) and anodization are the two main surface modification techniques which are used for most of the available implants systems in the market. Anodization is an electrochemical treatment of the implant surface, where the thickness of the titanium oxide layer is increased using an electrolytic process [15]. New topography, chemistry, and degree of crystallinity of the implant surface after anodization depend on the amount of time and level of voltage during the electrolytic process. In the SA process, small hard ceramic particles such as Al_2_O_3_ and TiO_2_ or calcium phosphates are used to blast and create craters on the surface of machined implants (see Figure 1(a)). These particles range in size from 10 to several 100 *μ*m and are projected at a high velocity through a nozzle by compressed air or a fluid [16]. Following blasting, the implants are immersed in a strong acid (e.g., HCl, H_2_SO_4_, HF, or HNO_3_) solution at elevated temperatures (see Figure 1(b)) to remove remnant blasting particles attached or wedged to the surface and to further alter the surface topography and chemistry. Surface properties of SA-modified surfaces are dependent on several manufacturing parameters, such as the size of the blasting particles, the velocity of particles and blasting duration, the composition of the acid solution used for cleaning after blasting, and the time and temperature of exposure to the acid solution. Anodization and SA process aim to increase the roughness of machined titanium from initial area roughness average (S_a_) of ≤0.5 *μ*m to S_a_ of 1−2 *μ*m. A recently published systematic review on studies reporting on long-term survival of implant systems with different surfaces showed that both anodization and SA processing of implant surfaces improved the survival rate of implants: 98.5% by anodization and 96.7% by SA, compared to 96.4% for machined surface implants after 10 years or longer in function [5].

One aspect of titanium surface modification that continues to garner increasing attention is how a modification process and its control affect the cleanliness of resulting implant surface [17]. There are studies showing presence of organic and inorganic contaminations on the surface of different sterile-packaged implant systems on the market [17, 18]. The anodization process in itself does not lend to particle contamination. However, investigating the presence of remnant particles on a moderately rough surface is relevant in the case of surfaces modified with the SA process, as the roughness is created by bombarding with particles that may potentially remain on the implant surface even after acid-etching [18–21]. Despite the large number of studies to investigate the surface properties of commercially available implant systems, a comprehensive study on the cleanliness of SA-modified implants systems is not yet available.

In this investigation, the quantity and distribution of blasting particle remnants on the surface of nine commercially available major implant systems produced by seven different manufacturers according to the SA process were evaluated in order to assess whether manufacturing has any impact on the surface cleanliness and reproducibility of a particular surface topography.

## 2. Materials and Methods

### 2.1. Samples

Nine commercially available implant systems produced by seven manufacturers using a SA process were analyzed: SPI NanoTec™ (Alpha-Bio Tec Ltd, Israel), Conelog® Promote® (Camlog Biotechnologies AG, Switzerland), Ankylos® Friadent plus® (Dentsply Sirona Dental GmbH, USA), ProActive® Straight Implant (Neoss Ltd, UK ), USIII CA Fixture (Osstem Implant Co Ltd, USA), BLX Roxolid® SLActive® (Institute Straumann AG, Switzerland), Bone Level (BL) Roxolid® SLA® (Institute Straumann AG, Switzerland), BL Roxolid® SLActive® (Institute Straumann AG, Switzerland), and SPI®ELEMENT INICELL® (Thommen Medical AG, Switzerland). Implant lots were purchased between September 2018 and January 2019. Six implants selected from at least 3 different lots were analyzed for each implant system (total of 54 implants). The information on diameter, length, and lot of the investigated implants is presented in Table 1.

### 2.2. Scanning Electron Microscopy (SEM) Analysis

Implant surfaces were analyzed by SEM using a Zeiss Supra® 40 VP microscope (Oberkochen, Germany). The implants were carefully fixed in a clamp holder without touching their surface. Implants packed and delivered in a storage solution, i.e., Straumann implants with SLActive® surface, were rinsed before fixation in the clamp holder for 30 seconds with deionized water and dried with nitrogen. The implants were examined without surface sputtering. Images were acquired with an acceleration voltage of 20 kV using a backscatter imaging detector.

**Figure 2:**
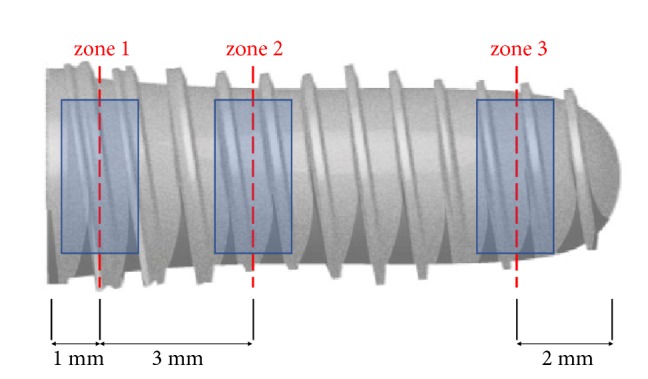
Zones and regions of interest (ROI) for particle count measurement defined for study purposes. Three zones (red dash-line) were selected at 1 mm and 4 mm from the collar and at 2 mm from the apex each. For each zone, two ROIs (blue rectangle) were analyzed at opposite sides of the implant (6 ROIs per implant).

Three zones were defined to evaluate the surface at the same position for all implants (see Figure 2): zone 1 (1 mm from the collar in the apical direction); zone 2 (4 mm from the collar in the apical direction); and zone 3 (2 mm from the apex in the coronal direction). Following the evaluation of the three zones, each implant was rotated by 180 degrees around the long implant axis and the evaluation repeated for the corresponding zones on the opposite side. Overview images at a magnification of x64 were captured in three regions of interest (ROI) corresponding to the three zones. ROI was a rectangle of 3 mm × 1.5 mm, except for zone 3 in SPI NanoTec™ and BLX Roxolid® SLActive® implants, in which the ROI was measured to be 2.046 mm × 2.2 mm due to the narrow apex of the implants. Consequently, each ROI was a rectangle of 4.5 mm^2^.

The SEM backscatter electron detector was used to quantify the number of particles in each ROI. Particles were marked and numbered. The size of each particle was measured using IMS software (Imagic Imaging Ltd., Glattbrugg, Switzerland) and particles smaller than 10 *μ*m were excluded from quantification. Additionally, an image of each particle was taken and its elemental composition was determined using energy-dispersive X-ray spectroscopy (EDX) with backscatter electron detector.

### 2.3. Statistical Analysis

Statistical analyses were performed using R 3.5.1 (R Foundation for Statistical Computing, https://www.R-project.org) with algorithms based on standard libraries. Analysis of Variance (ANOVA) modelling was used to compare particle counts and particle sizes based on the implant brand, lot, and zone of measurement. Fitted ANOVA values of implant brands were compared using Tukey's Honest significance test (family-wise significance level of 0.95). Variances were compared using two-sample F-tests.

## 3. Results

### 3.1. Presence of Particles on the SA-Modified Implant Surface

**Figure 3:**
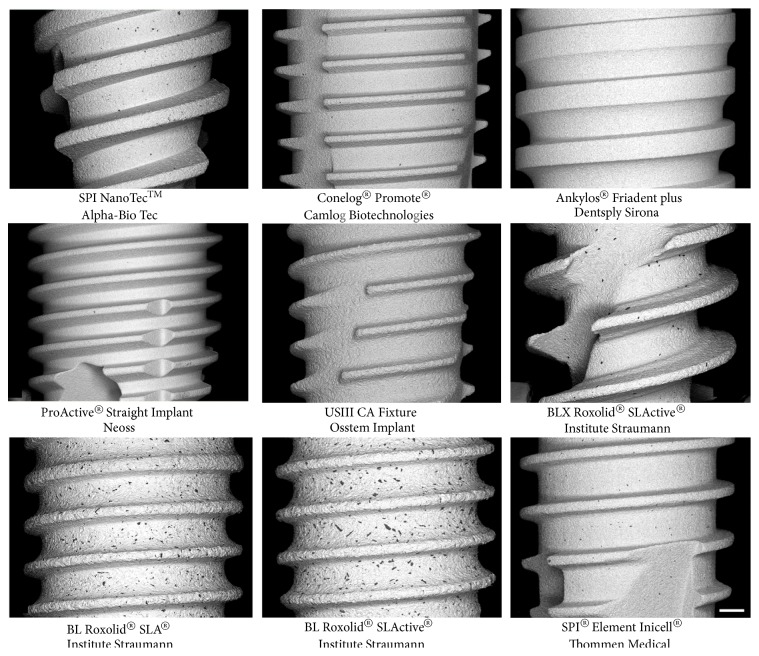
Backscatter-SEM micrographs of the analyzed implant systems. Note the various numbers and sizes of the Al_2_O_3_ particles (black dots) remaining on the surface following etching. Scale bar: 200 *μ*m.

**Figure 4:**
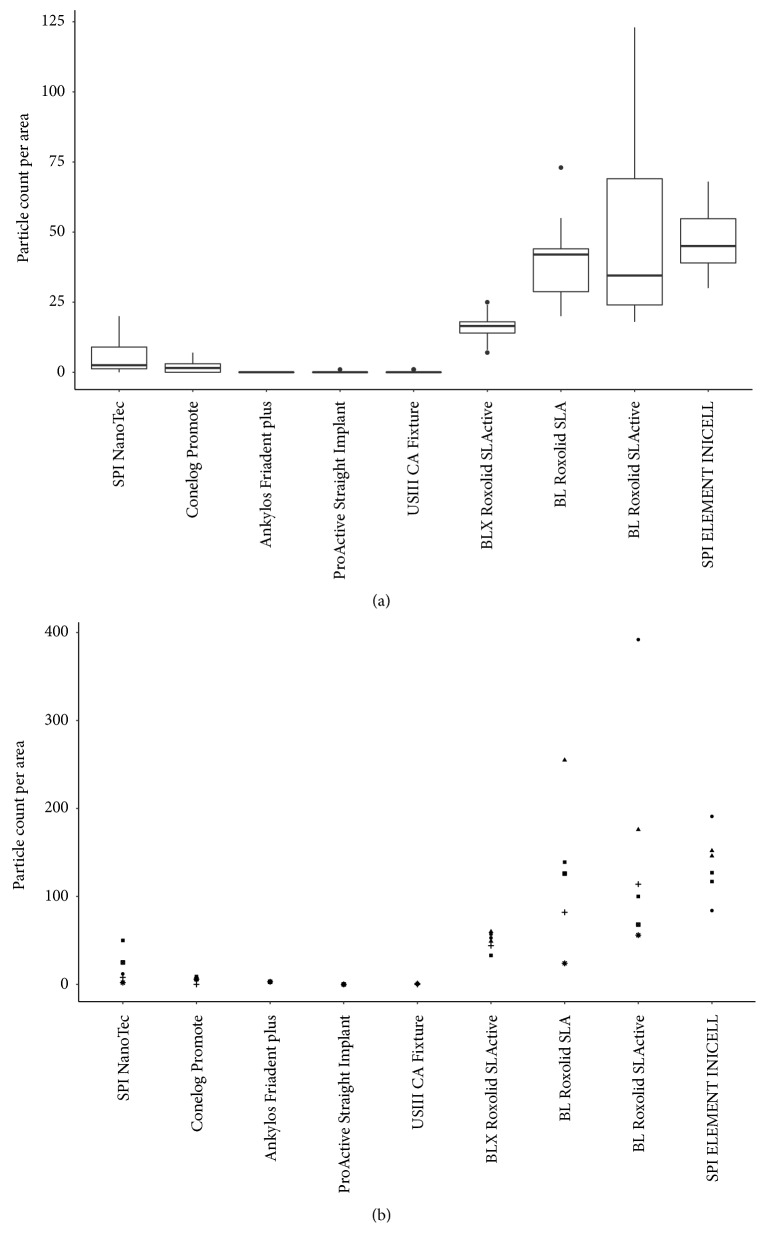
Particle count variation in 9 implant systems: (a) box plot of surface particle counts in 9 implant systems; (b) variations in surface particle counts; different markers indicate implants of different lots.

SEM showed presence of remnant particles on all tested implant surfaces, with the exclusion of Ankylos® Friadent plus® (see Figure 3). The mean counts of remnant particles in the six zones per implant varied between different implant systems. The mean particle counts were higher for surfaces manufactured by Thommen Medical and Institute Straumann compared with the other implant systems (see Figure 4(a) and Table 2). According to the ANOVA model, the difference between particle counts on SPI®ELEMENT INICELL®, BL Roxolid® SLActive®, BL Roxolid® SLA®, and BLX Roxolid® SLActive® implant surfaces and the other tested implant systems was statistically significant (all p<0.05), with the exception of the difference between BLX Roxolid® SLActive® and SPI NanoTec™ (p=0.20).

### 3.2. Variation in Particle Counts

Variation in the counts of remnant particles was evaluated for a combination of implants from the same or different manufactured lots for each implant system. Implant systems BL Roxolid® SLActive® and BL Roxolid® SLA® showed a higher variation in particle counts compared with those in other implant systems (see Figure 4(a)). Moreover, BL Roxolid® SLActive® and BL Roxolid® SLA® implant surfaces obtained from the same lot displayed a high degree of variation in particle counts (see Figure 4(b)). According to two-sample F-tests used for pairwise comparisons, variances of particle count were significantly different for all implant systems (all p<0.05), with the exclusion of SPI NanoTec™ vs. BLX Roxolid® SLActive® (p=0.56), ProActive® Straight Implant vs. USIII CA Fixture (p=0.05), and SPI®ELEMENT INICELL® versus BL Roxolid® SLA® (p=0.27).

### 3.3. Elemental Composition, Morphology, and Size of the Particles

**Figure 5:**
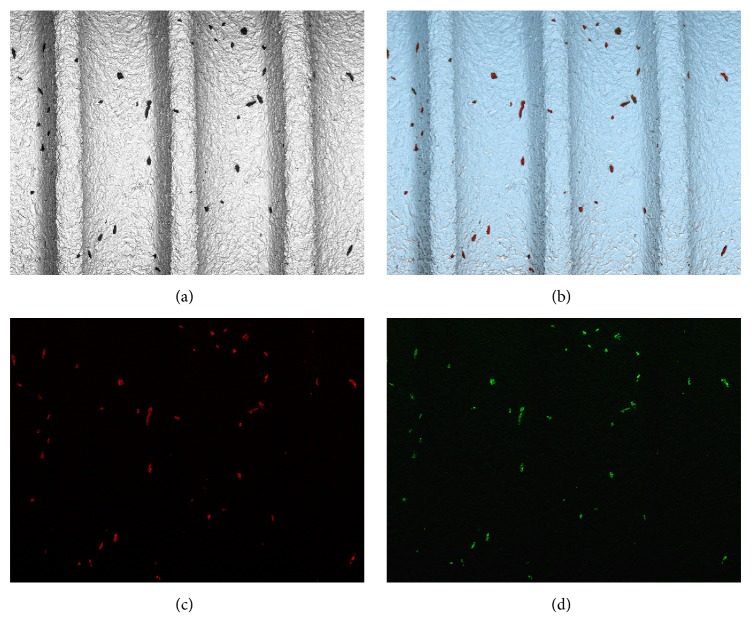
Micrographs of BL Roxolid® SLActive® implant surface (a), overlay of elements Al and O (b), and mapping of elements Al (c) and O (d).

**Figure 6:**
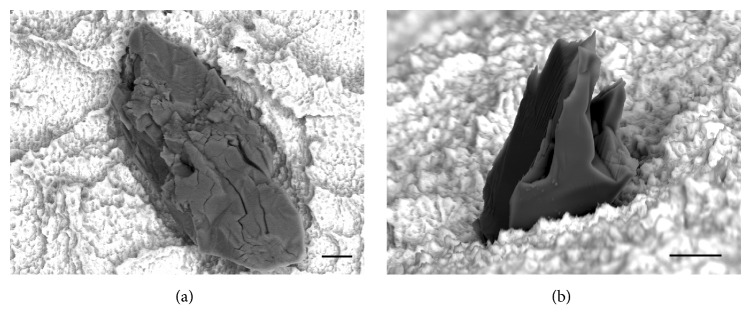
High magnification images of Al_2_O_3_ particles. Scale bar: 10 *μ*m. Note the cracked morphology of the particle on the surface of BL Roxolid® SLActive® implant in (a) and a particle protruding out from the surface of BLX Roxolid® SLActive® implant (b). Scale bars: 10 *μ*m.

**Figure 7:**
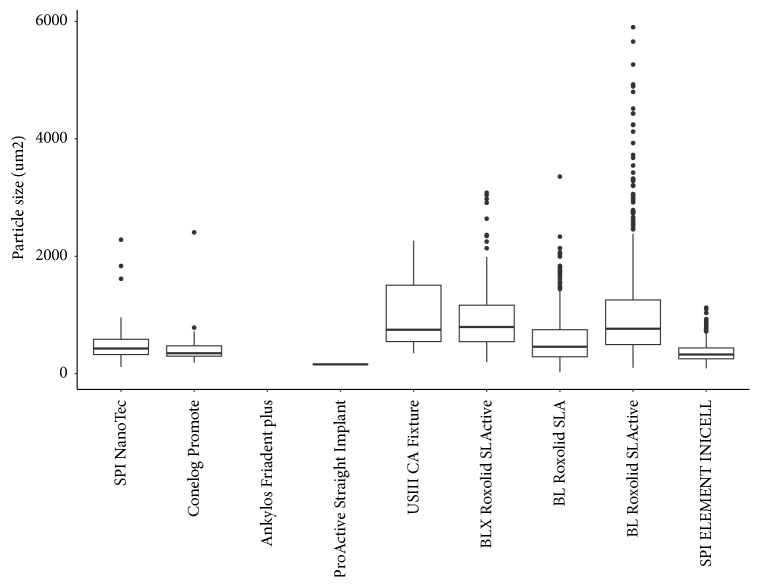
Box plot of particle size on 9 implant systems.

EDX analysis showed that the particles were composed of Al and O (see Figure 5), suggesting that they were Al_2_O_3_ particle remnants of the blasting process. Most particles revealed a brittle and cracked morphology (see Figure 6(a)) and were protruding from the surface up to 30 microns (see Figure 6(b)). Mean size of the particles varied from 159 to 1120 *μ*m^2^, with particles remaining on BL Roxolid® SLActive® implant showing an individual size of up to 5900 *μ*m^2^ (see Table 2 and Figure 7).

## 4. Discussion

**Figure 8:**
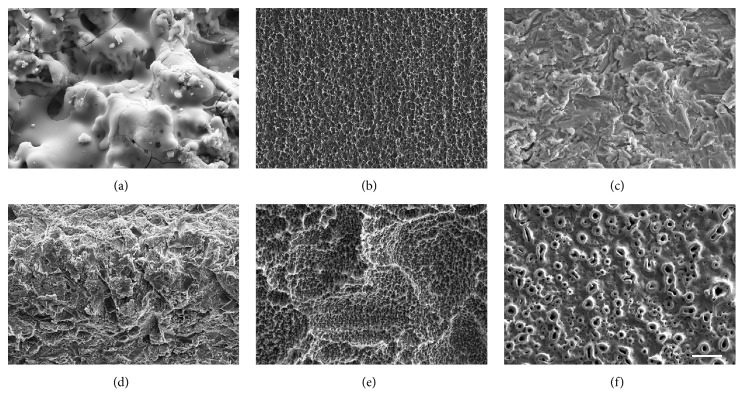
SEM micrographs of different moderately rough implant surfaces in the market: (a) hydroxyapatite coated, (b) acid etched, (c) Ca_3_(PO_4_)_2_ blasted, (d) TiO_2_ blasted, (e) sandblasted and acid-etched, and (f) anodized. Scale bar: 20 *μ*m.

Dental implants with moderately rough surfaces osseointegrate faster and their use has significantly decreased early failures and enabled application of immediate loading protocols [4, 5, 10]. Different dental implant manufacturers have developed different techniques to achieve moderate roughness, including titanium plasma spraying, coating with hydroxyapatite, sandblasting, acid-etching, laser ablation, sandblasting combined with acid-etching (SA), and anodization (Figure 8).

Since its introduction in the early 1990s [22], the SA process has become widely used by several implant manufacturers. However, because this manufacturing process involves bombarding the implant surface with particles that may potentially remain on the implant surface even after acid-etching, it is possible that the resulting products contain remnant particle contamination [18–21].

The results of the current analysis on 9 major dental implant systems modified with the SA process demonstrate that most surfaces of sterile-packaged, commercially available dental implants contain particle contamination. EDX analysis demonstrated that the detected particles were Al_2_O_3_, indicating that they were remnants from the blasting step of the manufacturing process. These results confirm the findings of a previously published study, which showed that Al_2_O_3_ particles might cover up to 14.4% of the implant surface [18].

Overall, the present study reveals that the analyzed titanium dental implants do not have similar surface properties, even though they were all created using the SA process. Lack of adequate control over the blasting and acid etching process appears to result in blasting particles being wedged into the surface of the implant and not being fully removed during acid etching. In consequence, the final product may contain high numbers of blasting particle remnants, as detected for investigated Straumann BL and BLX implants as well as for Thommen INICELL implant line. Moreover, the low count of particle remnants observed in other implant systems manufactured by, e.g., Dentsply Sirona suggested that the SA manufacturing process can be controlled to achieve minimal levels of particle remnants.

Variation in the count of remnant particles also differed within manufacturer and their implant lines, with the BL Roxolid® SLActive® surface showing the highest variation, even when the assessment was made on implants originating from the same manufacturing lot. This finding supports our hypothesis that the SA manufacturing parameters and process control are not similar across brands.

Furthermore, the size of the remnant particles was different. The largest measured size of a particle was 5900 *μ*m^2^ for BL Roxolid® SLActive® implant system, and the largest mean was 1120±1011 *μ*m^2^ measured for USIII CA Fixture. SEM analysis revealed that the blasting particle remnants have a brittle, cracked morphology and are protruding from the surface, suggesting an unstable arrangement of these particles on the implant surface. One might expect that, due to their partial inclusion to respective surface, these particles may become dislodged and migrate during implant insertion. This hypothesis is supported by a recent study showing that the surface roughness of the SLA implant system decreases significantly after insertion [21, 23]. The clinical relevance of the remnant particles is unknown today; however, possible local or systemic adverse effects of this contamination cannot be fully excluded. Because surface contamination can be avoided in the production process as shown by some manufacturers, clinicians should be able to expect a clean implant surface when treating their patients.

## 5. Conclusions

Implant surface quality cannot be assessed by visual inspection. Clinicians must be able to trust the implant manufacturer that the manufacturing process was appropriately designed and adequately controlled so that the final product meets their quality expectations. This study revealed that not all manufacturers provide such quality assurance. The findings highlight that adequate process control over surface modification using the SA technique is paramount for achieving a clean and consistent final medical device prior to placement in a patient.

## Figures and Tables

**Table 1:** Implant size (ø [mm]x length [mm]) and lot number for 6 tested implant specimens per each implant system.

Implant manufacturer	Alpha-BioTec	Camlog Biotechnologies	Dentsply Sirona	Neoss	Osstem	Institute Straumann			Thommen Medical

Specimen	Implant system								
	SPI NanoTec™	Conelog® Promote®	Ankylos® Friadent plus®	ProActive® Straight Implant	USIII CA Fixture	BLX Roxolid® SLActive®	BL Roxolid® SLA®	BL Roxolid® SLActive®	SPI®ELEMENT INICELL®

Specimen 1	4.2 x 10.0	4.3 x 9.0	4.5 x 9.5	4.0 x 9.0	4.0 x 10.0	4.5 x 10.0	4.1 x 10.0	4.1 x 10.0	4.5 x 9.5
	17188537	78276	B180004244	23229	FUP17A053	PW721	NZ528	PM914	17983
Specimen 2	4.2 x 10.0	4.3 x 9.0	4.5 x 9.5	4.0 x 9.0	4.0 x 10.0	4.5 x 10.0	4.1 x 10.0	4.1 x 10.0	4.5 x 9.5
	18091176	81279	B170017324	23921	FUP18C008	PW721	RJ536	RK729	17029
Specimen 3	4.2 x 11.5	4.3 x 11.0	4.5 x 11.0	4.0 x 11.0	4.0 x 11.0	4.5 x 12.0	4.1 x 12.0	4.1 x 12.0	4.5 x 11.0
	17128176	78624	B180002597	23832	FUP17A055	PW722	PM658	PZ373	16987
Specimen 4	4.2 x 11.5	4.3 x 11.0	4.5 x 11.0	4.0 x 11.0	4.0 x 11.0	4.5 x 12.0	4.1 x 12.0	4.1 x 12.0	4.5 x 11.0
	18106872	81025	B170016078	23832	FUP18E019	PW722	RG324	PK477	17911
Specimen 5	4.2 x 13.0	4.3 x 13.0	4.5 x 14.0	4.0 x 13.0	4.0 x 13.0	4.5 x 14.0	4.1 x 14.0	4.1 x 14.0	4.5 x 12.5
	17077554	77240	B180000617	23334	FUP18E058	RA955	MN169	KT049	18030
Specimen 6	4.2 x 13.0	4.3x 13.0	4.5 x 14.0	4.0 x 13.0	4.0 x 13.0	4.5 x 14.0	4.1 x 14.0	4.1 x 14.0	4.5 x 12.5
	18079733	78279	B170014366	23338	FUP17B033	RA929	RA926	NZ636	18030

**Table 2:** Quantification of remnant particle counts and sizes in the ROIs.

Implant manufacturer	Alpha-BioTec	Camlog Biotechnologies	Dentsply Sirona	Neoss	Osstem	Institute Straumann			Thommen Medical

Implant system	SPI NanoTec™	Conelog® Promote®	Ankylos® Friadent plus®	ProActive® Straight Implant	USIII CA Fixture	BLX Roxolid® SLActive®	BL Roxolid® SLA®	BL Roxolid® SLActive®	SPI®ELEMENT INICELL®

Particle count									
Mean ± SD	5.3 ± 5.7	1.7 ± 1.9	0.0 ± 0.0	0.1 ± 0.2	0.2 ± 0.4	16.4 ± 5.0	38.9 ± 13.5	50.3 ± 35.7	46.6 ± 10.3
Range (min; max)	(0;13)	(0;6)	NA	(0;1)	(0;1)	(0;33)	(0;134)	(0;205)	(0;98)

Particle size									
Mean ± SD [*µ*m2]	508 ±318	457 ±398	NA	159	1120 ±1011	909 ±511	570 ±413	1002 ±781	357 ±147
Range (min; max)	(114; 2283)	(189; 2407)	NA	(159; 159)	(348; 2266)	(196;3082)	(26; 3357)	(101; 5902)	(91;1126)
n of particles	96	30	NA	1	3	296	752	905	839

## Data Availability

The raw data used to support the findings of this study are restricted due to commercial confidentiality. Data are available from Sebastian Bauer for researchers who meet the criteria for access to confidential data.
